# Procedural Parameters in Predicting Late Recurrence Following Catheter Ablation of Paroxysmal Atrial Fibrillation

**DOI:** 10.3390/jcm15062409

**Published:** 2026-03-21

**Authors:** Yangjing Xie, Xiaoxia Hu, Dongyu Ma, Ling Zhang, Ying Huang

**Affiliations:** Department of Cardiology, the First Affiliated Hospital of Anhui Medical University, Hefei 230032, China; xieyj@ahmu.edu.cn (Y.X.); yfy1141235@fy.ahmu.edu.cn (X.H.); dongyu_ma@fy.ahmu.edu.cn (D.M.); yfy264723@fy.ahmu.edu.cn (L.Z.)

**Keywords:** atrial fibrillation, contact force, ablation index, GAP, AF recurrence

## Abstract

**Background**: To investigate the predictive value of catheter ablation parameters during pulmonary vein isolation (PVI) on long-term recurrence in patients with paroxysmal atrial fibrillation (pAF). **Methods**: A retrospective analysis was conducted on 386 pAF patients who underwent initial catheter-based radiofrequency ablation (RFA) for PVI. After excluding ineligible cases and those lost to follow-up, 150 patients were included (mean follow-up: 28.86 ± 3.03 months). Patients were divided into recurrence and AF-free groups. Ablation parameters including catheter contact force (CF), ablation index (AI), and GAPs were collected via the CARTO VISITAG Module, and Cox regression was used to identify recurrence predictors. **Results**: The 2-year AF recurrence rate was 13.33% (20/150). No significant differences in baseline clinical characteristics, AI compliance rate, VISITAG GAP incidence, ablation points and time, or complication rates were observed between groups (*p* > 0.05). However, CF compliance rate was significantly lower in the AF recurrence group compared to AF-free group (79.17% vs. 90.10%, *p* < 0.001), and it was an independent predictor of late AF recurrence (HR = 0.950, 95%CI: 0.919–0.981; *p* = 0.002). **Conclusion**: CF compliance rate is independently associated with late AF recurrence after PVI. Maintaining stable CF during ablation may promote durable lesions and potentially reduce recurrence risk.

## 1. Introduction

Pulmonary vein isolation (PVI) is an effective treatment for paroxysmal atrial fibrillation (pAF) [1,2]. Catheter-based point-by-point radiofrequency ablation (RFA) is the most widely used technique and has proven to have a high success of AF-free rate [3,4]. However, the long-term outcome of AF recurrence remains suboptimal and frequently requires repeat ablation. Previous studies have indicated that the pivotal RFA parameters during the PVI procedure are closely associated with the AF recurrence, such as catheter contact force (CF) [5], catheter stability [6], ablation index (AI) [7], and the presence of GAP [8]. However, these studies are mostly performed relying on a single factor, lacking dynamic complexity [9,10]. In this study, we analyzed the initial RFA parameter data obtained from the CARTO VISITAG Module (Biosense Webster, Irvine, CA, USA), conducting a multivariate analysis to identify the key factors influencing the safety and efficacy of RFA in pAF patients.

## 2. Methods

### 2.1. Study Design

A retrospective analysis was conducted on 386 pAF patients who underwent initial catheter-based RFA for PVI between November 2022 and October 2023. All patients were followed up for rhythm outcomes and subsequently classified into a recurrence group or AF-free group based on AF recurrence. The study was approved by the local Ethics Committee.

The inclusion criteria include: (1) symptomatic pAF; (2) nonvalvular pAF; (3) age range 18 to 75 years; (4) without severe heart failure or severe hepatic and renal dysfunction. Any additional ablation of either the left or right atrium independently of PVI were excluded, such as additional linear ablation, substrate modification, superior vena cava ablation, paroxysmal supraventricular tachycardia ablation and ventricular arrhythmia ablation. Non-PV trigger provoked during the procedure was also excluded from the study.

### 2.2. AF Recurrence Follow-Up

Electrocardiography, Holter monitoring, or portable electrocardiogram devices were employed to perform continuous 24 h ambulatory electrocardiographic monitoring for AF recurrence. Atrial arrhythmia recurrence was defined as any electrocardiographically documented episode of AF, atrial tachycardia, or atrial flutter ≥ 30 s, occurring after the 3-month post-procedural blanking period and in the absence of Class I or III antiarrhythmic drug therapy.

### 2.3. PVI Procedure

PVI was performed under standard protocol by experienced operators. Briefly, pre-ablation evaluations, including routine blood test and transesophageal echocardiography (TEE), were performed for all patients. All procedures were performed under conscious sedation [11]. PVI was performed with irrigated catheter with contact force sensing (Thermocool Smart-Touch) under the CARTO 3 system. High-power RF ablation with energy setting 40–45 W was guided by the ablation index (AI) [12]. The AI target was set as 450–550 for the anterior wall, roof, floor, and ridge, and 350–400 for the posterior wall [13]. In cases where initial circumferential ablation did not result in complete PVI, supplementary point ablation (touch-up) was performed at identified gaps to ensure electrical isolation. Non-PV AF trigger was induced by burst pacing (cycle length 200–300 ms for 10 s) and isoproterenol (15–20 μg/min for 2 min). The ablation lesion was automatically tagged on the VISITAG Module. PVI was reconfirmed >20 min after its initial success. All patients received routine anticoagulation therapy for at least two months post-ablation.

### 2.4. Ablation Parameter Data Collection

The RF points in both groups were collected using the CARTO VISITAG Module V7. This software enables continuous recording, tracking and quantification of RF sites along with the parameters during RF applications. The VISITAG Module setting has been described previously [14]. RF sites were objectively annotated based on the following predefined criteria: average catheter tip-to-tissue contact force (CF) 10–20 g; average AI values: anterior wall 450–550, posterior wall 350–400; VISITAG GAP: maximum inter tag distance ≥ 6 mm.

### 2.5. Statistical Analysis

Statistical analyses were conducted by Statistical Package for the Social Sciences (SPSS) software (version 20.0). Continuous variables were presented as the mean ± standard deviation (mean± SD). Comparisons between groups were conducted using Student’s *t*-test for independent samples. Categorical variables were represented as relative frequencies, and comparisons were executed using χ^2^ tests. Statistical significance was set at *p*-value < 0.05. To identify independent predictive factors of AF recurrence following RFA, a multivariable Cox proportional hazards regression model was utilized. To avoid overfitting and to ensure model parsimony, covariates were prespecified based on clinical reasoning and the established literature. Furthermore, internal validation of the multivariable model was conducted using a bootstrapping approach to evaluate the consistency and stability of the estimated coefficients.

## 3. Results

### 3.1. Clinical Characteristics

**Figure 1:**
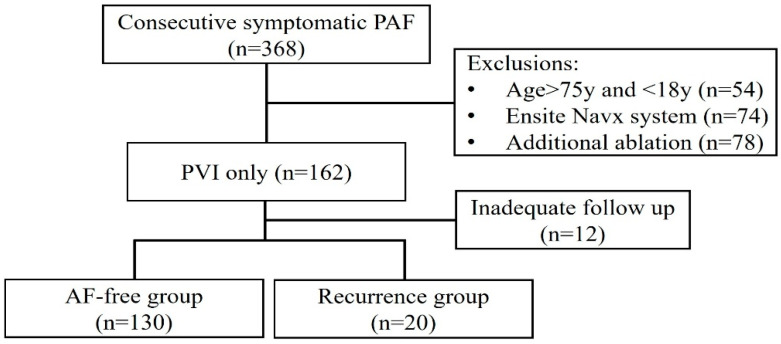
Study flow diagram. pAF, paroxysmal atrial fibrillation; PVI, pulmonary vein isolation.

A total of 386 pAF patients were screened, of whom 206 were excluded based on the exclusion criteria. The remaining 162 patients had received PVI only ablation and followed up for AF recurrence. In total, 12 out of 162 were lost to follow-up, and the mean follow-up duration was 28.86 ± 3.03 months. AF recurrence rate was 13.33% (20/150). Patients were subsequently assigned to the AF-free group (*n* = 130) or recurrence group (*n* = 20) according to the follow-up data (Figure 1).

The general clinical features of the patients in the two groups are summarized in Table 1. No statistically significant differences were observed in baseline characteristics between the two groups, including gender, age, body mass index (BMI), and comorbidities such as hypertension, diabetes, stroke/transient ischemic attack (TIA), and coronary artery disease (CAD). Additionally, there were no significant differences in left atrial (LA) size, left ventricular end diastolic diameter (LVEDD), ejection fraction (EF) or CHA2DS2-VASC score (Table 1).

### 3.2. Ablation Parameters and Complications

**Figure 2:**
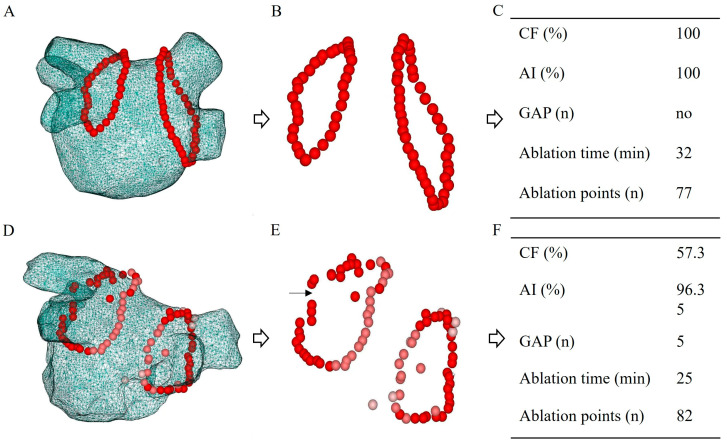
Ablation parameters recorded using the VISITAG system. The left atrium is shown from a posteroanterior view in panels A and D. CF and AI compliance rate were significantly higher in the AF-free group without GAP (**A**–**C**), while CF and AI compliance rate were considerably lower in the AF recurrence group with GAP (**D**–**F**). The narrow blank arrow points to the VISITAG GAP (**E**). CF, contact force; AI, ablation index.

Bilateral PVI was performed successfully in all patients (Figure 2). The compliance rates of ablation parameters were compared between the recurrence and AF-free groups. The AI compliance rate was 90.15% in the recurrence group and 89.73% in the AF-free group. The CF compliance rate was 79.17% in the recurrence group and 90.10% in the AF-free group. VISITAG GAPs were observed in 20.0% (4/20) of patients in the recurrence group and 12.30% (16/130) in the AF-free group. A statistically significant difference in CF compliance rate was observed between the two groups (*p* < 0.001); however, no significant differences were found for AI values or VISITAG GAP occurrence (Table 2).

In addition, the recurrence group underwent a mean of 85.00 ± 14.60 ablation points, compared to 80.41 ± 13.30 in the AF-free group. The average ablation duration was 28.05 ± 7.61 min in the recurrence group and 25.09 ± 6.13 min in the AF-free group. No procedural complications were observed in the recurrence group, whereas one case of pseudoaneurysm was reported in the AF-free group. However, no statistically significant differences were observed between the two groups with respect to ablation points, procedure time, or complication rates (Table 2).

### 3.3. Predictor of AF Recurrence

Univariate Cox regression analysis of all ablation parameters and baseline clinical factors revealed that CF compliance rate was significantly associated with AF recurrence (HR = 0.958, 95%CI: 0.933–0.982; *p* < 0.001). Multivariate COX regression analysis further demonstrated that the CF compliance rate was an independent predictor of AF recurrence over the 2-year follow-up period (HR = 0.950, 95%CI: 0.919–0.981; *p* = 0.002) (Table 3). The stability of this model was further confirmed by bootstrap analysis, which yielded a significant association for CF compliance (*p* < 0.001; 95%CI: −0.098 to −0.026), indicating that the model possesses high predictive reliability.

## 4. Discussion

Catheter-based PVI is a well-established treatment for patients with pAF [15]; however, the electrical reconduction in PVs is considered to be the major mechanism of AF recurrence [16,17,18]. Ablation parameters during the procedure, such as AI, CF and continuity of RF applications, are not only associated with lesion durability but also with the occurrence of complications.

AI is a quantitative, formula-based parameter for use in catheter ablation procedures for AF. AI incorporates CF, ablation time and power and has been shown to accurately estimate lesion depth [7]. Although AI-guided ablation has been widely used and has demonstrated consistent safety in the procedure of PVI, the association between AI target and AF recurrence remains unreported [12,19]. It is widely observed that lower CF, combined with longer ablation time, is often used to achieve the target AI during PVI procedures, particularly by less experienced operators. A well-defined target AI range is essential to ensure both the efficacy of ablation lesions and procedural safety. In this study, we found no statistically significant difference in AI complacence rate between the recurrence group and the AF-free group, suggesting that achieving a target AI does not substantially influence the risk of arrhythmia recurrence. Power constitutes another parameter in RF ablation. Recent evidence indicates that high-power, short-duration (HPSD) ablation protocols are associated with a higher rate of first-pass pulmonary vein isolation and reduced total ablation time [20]. However, the efficacy of HPSD ablation is contingent upon consistent, stable CF. Suboptimal contact may compromise lesion formation despite elevated power settings, thereby diminishing procedural efficacy and increasing the risk of procedural complications.

Several factors may influence CF stability, including atrial size, the location of transseptal puncture, patient’s cooperation, and the ablation site within the PVs. It is crucial to achieve optimal catheter CF prior to RF energy delivery to ensure effective lesion formation and procedural safety. CF has been recognized as a key determinant influencing ablation lesion and achieving the effective AI [21,22], and it had also demonstrated that maintaining an adequate CF level (target 20 g, range 10–30 g) is associated with the creation of durable PVI efficiency [10,23,24]. In addition, remapping studies have also demonstrated that CF was a strong predictor of electrical reconnections of PV and is considered the primary contributor to AF recurrence [24,25]. However, comprehensive multivariable analyses adjusting for potential confounding factors have been limited. In our study, we retrospectively compared the CF compliance rate between patients from AF-free and recurrence groups and found a significantly higher CF compliance rate in AF-free patients. Moreover, multivariate COX regression analysis revealed that CF is an independent predictor among ablation parameters for the late recurrence of pAF. Overall, CF has been recognized as a key determinant in maintaining long-term freedom from AF following catheter ablation. Maintaining CF within 10–20 g, ensuring ≥80% compliance range, significantly enhances both procedural efficacy and clinical applicability. While traditional metrics focus on mean CF or AI values, our study highlights the clinical superiority of the CF compliance rate. This VISITAG-derived parameter uniquely quantifies the consistency and stability of catheter–tissue contact rather than average metrics. By prioritizing the uniformity of lesion formation over intermittent high-pressure peaks, CF compliance provides a more reliable predictor for preventing electrical reconduction and ensuring long-term rhythm stability.

Discontinuous ablation lines, despite exhibiting acute isolation, may have propagation recovery over time, allowing the recurrence of arrhythmias [26]. A recent redo AF ablation trial also showed that PV GAPs alone were associated with recurrence. These studies defined GAP as an electrical reconnection narrow zone identified during redo procedures, where there was possibly an ablated lesion initially. It is important to note that the “VISITAG gaps” in our analysis were defined by a geometric distance of ≥6 mm between adjacent ablation sites. This is distinct from the electrical reconnections identified during redo procedures. Since acute PVI was achieved in all subjects, these anatomical GAPs did not translate into immediate electrical conduction. Consequently, no significant correlation was observed between VISITAG GAPs and late AF recurrence in this cohort. However, this conclusion should be interpreted with caution, as the functional significance of these geometric GAPs requires further validation through electrophysiological remapping.

In addition to ablation-related parameters, several risk factors influence AF recurrence [27], including age [28], alcohol consumption [29] and hypertension [30]. In the present study, to minimize the potential confounding effects of age, patients aged >75 years were excluded. Hypertension is the most prevalent comorbidity among patients with AF. Chronic pressure overload may promote the development of AF by inducing structural and electrical remodeling of the left atrium, while the underlying molecular mechanisms are not fully understood. Moreover, obesity and alcohol consumption are well-established risk factors for both the onset and recurrence of AF. In the present study, all AF patients underwent comprehensive postoperative management, including strict blood pressure control, weight management strategies, and alcohol abstinence. These interventions may play a critical role in enhancing the overall success rates of treatment. Beyond lifestyle modifications, emerging pharmacological strategies, particularly the use of SGLT2 inhibitors (SGLT2i), have shown promise in reducing post-ablation AF recurrence. Recent evidence suggests that SGLT2i may exert significant antiarrhythmic and pleiotropic effects, including the attenuation of atrial remodeling, which could further improve long-term rhythm control [31]. Integrating such therapies into post-procedural management represents a vital area for future research.

Meanwhile, the procedure-related complication reported in this study was one case of pseudoaneurysm, with no severe complications such as pericardial effusion or tamponade. This favorable safety profile may be attributed to the relatively preserved general condition of the pAF patients, as well as the use of intracardiac ultrasound (ICE) for the guidance of transseptal puncture and continuous pericardial monitoring throughout the procedure.

Furthermore, left atrial (LA) structural and functional remodeling is a well-established determinant of AF recurrence. In this study, we utilized the left atrial diameter (LAD) as the primary indicator of LA size. While the LA Volume Index (LAVI) is often considered a more comprehensive metric, we noted that volumetric measurements derived from three-dimensional modeling can be susceptible to geometric assumptions and may not always yield consistent anatomical representations across all patients. Given the retrospective nature of this cohort, LAD provided a highly reproducible and standardized clinical baseline. Nevertheless, the integration of advanced functional indices, such as LA strain, remains a promising direction for future research to further refine the predictive models of post-ablation outcomes.

This study has several limitations. First, the relatively small sample size may limit the generalizability of the findings. Future research should include larger, more diverse samples to enhance the robustness and external validity of the results. Second, the absence of electrophysiological confirmation in patients with recurrence constrains the understanding of the underlying recurrence mechanisms. Further electrophysiological assessments would provide more objective evidence and contribute to a clearer elucidation of the pathophysiological processes involved.

## 5. Conclusions

This retrospective study suggests that CF compliance rate is associated with late recurrence of AF following catheter ablation. Our findings indicate that maintaining stable catheter contact may contribute to more durable lesion formation, potentially enhancing long-term rhythm control in patients with pAF.

## Figures and Tables

**Table 1:** Patient demographics.

	Recurrence Group (*n* = 20)	AF-Free Group (*n* = 130)	*p*-Value
Age (y)	61.35 ± 11.85	58.16 ± 10.58	0.219
Male, N (%)	12 (60)	64 (49.23)	0.370
LAD (cm)	4.03 ± 0.64	3.95 ± 0.46	0.489
LVEDD (cm)	4.88 ± 0.34	4.81 ± 0.40	0.482
LVEF (%)	61.75 ± 4.52	61.00 ± 4.30	0.473
BMI (kg/m^2^)	24.70 ± 3.47	24.46 ± 3.26	0.766
Drinking, N (%)	2 (10.00)	28 (21.17)	0.230
Smoking, N (%)	2 (10.00)	35 (22.35)	0.102
CHA2DS2-VASc score	1.90 ± 1.37	1.70 ± 1.36	0.559
Comorbidities, N (%)			
Hypertension	11 (55.00)	53 (40.76)	0.231
Diabetes	2 (10.00)	18 (13.84)	0.638
Stroke/TIA	4 (20.00)	14 (10.76)	0.237
CAD	1 (5.00)	13 (10.00)	0.474

Continuous variables were presented as the mean ± SD. Comparisons between groups were conducted using Student’s *t*-test for independent samples. Categorical variables are represented as relative frequencies, and comparisons were executed using χ2 tests. Abbreviations: LAD, left atrial diameter; LVEDD, left ventricle end diastolic diameter; LVEF, left ventricular ejection fraction; BMI, body mass index; TIA, transient ischemic attack; CAD, coronary artery disease.

**Table 2:** Procedural parameters during ablation procedure.

	Recurrence Group (*n* = 20)	AF-Free Group (*n* = 130)	*p*-Value
Contact force (%)	79.17 ± 16.83	90.10 ± 6.53	<0.001
AI (%)	90.15 ± 9.17	89.73 ± 8.09	0.831
GAP, N (%)	4 (20.00)	16 (12.30)	0.346
Total ablation points (*n*)	85.00 ± 14.60	80.41 ± 13.36	0.160
Total ablation time (min)	28.05 ± 7.61	25.09 ± 6.13	0.054
Complications, N (%)	0 (0.00)	1 (0.76)	0.694

Abbreviations: AI, ablation index; AF, atrial fibrillation.

**Table 3:** Factors associated with AF recurrence after ablation.

	Univariate		Multivariate	
	HR (95% Cl)	*p*-Value	HR (95% Cl)	*p*-Value
Baseline factors				
Age	1.023 (0.979–1.069)	0.313	0.986 (0.936–1.039)	0.600
LAD	1.969 (0.777–4.990)	0.153	2.075 (0.633–6.798)	0.228
Drinking	2.258 (0.522–9.765)	0.276	1.718 (0.380–7.757)	0.483
Hypertension	0.635 (0.261–1.544)	0.316	0.843 (0.295–2.407)	0.750
Ablation factors				
Contact force	0.958 (0.933–0.982)	<0.001	0.950 (0.919–0.981)	0.002
AI	0.991 (0.942–1.042)	0.714	1.019 (0.965–1.077)	0.496
GAP	0.477 (0.156–1.453)	0.192	0.557 (0.165–1.880)	0.346

Hazard ratios and 95% CI were calculated using a Cox regression analysis. Abbreviations: LAD, left atrial diameter; AI, ablation index; AF, atrial fibrillation.

## Data Availability

The original contributions presented in this study are included in the article. Further inquiries can be directed to the corresponding authors.
